# Round the Hospitals

**Published:** 1918-11-02

**Authors:** 


### ROUND THE HOSPITALS.

THE KING held an Investiture in the Ball-room of Buckingham Palace on October 24, when four V.C.s were conferred, and many distinguished officers, among whom were a few nursing sisters, were introduced into the presence of the King and were invested by him with the insignia of the respective divisions of the Orders into which they have been admitted or decorated for bravery in the field. Owing to the proceedings taking place in the Palace instead of in the open air, it was found impracticable to admit members of the public or recipients' friends. The scene was very impressive, and will not soon be forgotten by those privileged to be present. The following were admitted Associates of the Royal Red Cross:—Sister Isobel Whyte, Q.A.I.M.N.S.; Sisters Sarah Bowe, Annie Duncan, and Frances Spedding, Q.A.I.M.N.S.(R.); Sister Helen Drinkwater, T.F.N.S.; Sister Annie Walker,

**ROUND THE HOSPITALS—(*continued*).**

R.R.C.S.; Miss Muriel Batey; Mrs. Frances Crawshaw; and Miss Hester Trimble, V.A.D. The Military Medal was awarded to Miss Muriel Thompson, First-Aid Nursing Yeomanry.

THE House of Commons voted to admit women members without reluctance or dismay. There was little, if any, lobbying, no feverish excitement. The cause is won so far, for it is hardly probable that the Lords will register a protest. It remains for the first woman to secure election. We are justified in expecting that representatives of the Nursing Service will offer themselves as candidates at the next election, and shall be surprised if they do not prove popular with the electors. As the next Parliament will undoubtedly be called on to lend serious attention to a Bill for the State Registration of Nurses, the importance of having wide-minded women in the House acquainted with all the facts under discussion cannot be over-estimated.

AN important step has been taken by the War Office in reconstituting the two advisory boards which up till now have dealt respectively with Queen Alexandra's Imperial Military Nursing Service and the Territorial Force Nursing Service. A new joint board is to take their place, under the title of Queen Alexandra's Army Nursing Board, with Her Majesty Queen Alexandra as President. The joint board has been formed to secure greater uniformity of administration in the two Services, which themselves remain distinct as heretofore. The urgent questions now under consideration with regard to coming demobilisation of the Nursing Services render it most desirable that uniformity should be secured in advance. The joint board consists of the Director-General, Army Medical Service (chairman), the Matrons-in-Chief of the two Nursing Services, a representative of the Voluntary Aid Detachments, Lady Ampthill, G.B.E., C.I.; six lay women, viz. the Dowager Countess of Airlie (vice-president); the Countess Roberts, O.B.E.; the Countess of Minto, C.I.; Lady Codrington; Lady Knox; Miss E. S. Haldane, LL.D.; and six matrons of civil hospitals, viz. Miss Cox-Davies, R.R.C., Royal Free Hospital; Miss G. Lloyd Still, C.B.E., R.R.C., St. Thomas's Hospital; Miss A. B. Baillie, R.R.C. (Principal Matron, T.F.N.S.), Royal Infirmary, Bristol; Miss H. Gregory Smith, R.R.C., Western Infirmary, Glasgow; Miss E. S. Innes, R.R.C., General Infirmary, Leeds; Miss M. G. Montgomery, the Middlesex Hospital.

ST. BARTHOLOMEW'S HOSPITAL is going through a bad time with the influenza. Four out of the resident medical staff of eighteen are under treatment for the disease, and five students. One of the students has died of it. Thirty-three nurses have taken the infection, and twenty-two are still off duty. Many serious cases from outside have been received into the wards. At Tottenham the Prince of Wales's Hospital is said to be "choked with influenza patients." The Beckenham Cottage Hospital has been severely visited. The matron, three nurses, and the entire domestic staff, in addition to six of the patients, were attacked. Only one case has proved fatal. Medical testimony is very strong on the fact that the highest rate of mortality is among persons between the ages of twenty-five and forty-five, who try to follow their occupation with a high temperature. Nurses are very much exposed to the temptation to bluff the disease, and would serve the interests of their patients better by succumbing for a few days in the early stages instead of waiting till they are too ill to move.

LADY FRANCES BALFOUR, in a letter to the press, brings to light the wretched condition of ships' passengers during an outbreak of influenza. In one case of which she has cognisance there were neither medicines nor medical attendance, but the situation was saved by "certain women nurses not on duty," who gave devoted service to the sufferers and saved the lives of some of those on board. "It was done by sheer will-power and determination" was the word of one who saw the work and the untoward conditions. Lady Balfour hints that these passengers voyaging totally unprovided with aid in sickness were soldiers. If so, someone has blundered again pretty badly.

THE Ministry of Labour is instituting a scheme for bringing recreation within reach of women war-workers in the various corps—Queen Mary's, the Wrens, and the Air Service. The Queen has graciously consented to attend the first invitation performance, which will be a sample of the programmes to be carried out in the camps, hostels, and other centres of women's war work. The idea is for the women belonging to the various corps to organise the entertainments for themselves; there will be competitions in singing and music, drawing, design, working banners, acting, dancing, sports, handicrafts—a good model for some of the Centres of the College of Nursing. What war-workers are more in need of the stimulus of such organised entertainment than nurses?

WE have heard no worthier tribute to nurses than that rendered by Colonel T. Sinclair, C.B., A.M.S., in his recent address to students at the Royal Victoria Hospital, Belfast. He declared himself amazed at the courage, devotion, and endurance of the sisters and nurses at the Front. They would nurse in "No Man's Land," if the Army chiefs would allow it. In trials of endurance they beat the men absolutely, always declining to rest, even for a moment, when on duty during operations. When their wards had been bombed they had stuck to their posts, soothed and dressed the doubly injured or shell-shocked soldiers, and assisted in the rescue of others. This had been beyond all praise, for in many cases it had been done by sisters wounded themselves, who had suppressed the fact till the following morning. Many sisters had responded to a call for anæsthetists, and, having undergone a three months' training, had admirably performed these new duties.

COLONEL SINCLAIR could not forbear a special word of appreciation of the sisters and nurses of the Royal Victoria Hospital and their matron, Miss Bostock, R.R.C. He repeated with pride that Miss Becher had told him she would like plenty of Royal Victoria nurses, and was able to record the award of four Royal Red Crosses, two Military Medals, and four mentions in dispatches to the sisters and nurses "so admirably trained" in the institution. We are glad that he had a word of admiration, too, for "the magnificent self-denial" of the nurses who had kept the home hospitals in full swing. Many will echo his hope that when rewards and honours are being distributed those who have kept the home fires burning will receive the full measure of honour for their splendid service at home.

Belfast has shouldered its responsibilities in respect of the Nation's Tribute to Nurses with zeal and satisfaction. A meeting was held in the City Hall on October 17. Lady Clanwilliam was in the chair, and an address was given by Mr. Harvey McLaughlin, honorary organising secretary of the movement. He stated that when Sir Arthur Stanley made his appeal for £100,000 an undertaking was made that Ireland would contribute £10,000 towards the Tribute Fund. Already from the different counties £8,000 had been subscribed, and they now looked to Belfast to raise £2,000 and complete the total promised. The money raised in Ireland is to be invested in Ireland under four trustees, and the interest is to be used on behalf of Irish nurses, partly to promote a system of insurance for nurses at low rates, and partly to give annuities to disabled nurses. The High Sheriff emphasised the enormous value of the work done by civilian nurses, who were scandalously underpaid, and the meeting pledged itself by acclamation to do their part in supporting the Nation's Tribute to Nurses.

Liverpool's College Centre opened its winter series of post-graduate lectures on October 23 with a very interesting address by Mr. T. C. Littler-Jones, F.R.C.S., on "The Surgical Nursing of Common War Injuries." The subject was one calculated to attract a large audience in so busy and important a hospital centre as Liverpool, and its interest was increased by the excellent demonstration which accompanied the lecture of up-to-date splints and appliances. A monthly lecture has been arranged up to April, to which members of the College are admitted free, and non-members for 3s. for the series. Further particulars of the lectures are given on page 102.

The Victoria League Club at Rutland Square, Edinburgh, for nurses from the British overseas Dominions and America, has done an excellent work during the six months of its existence, and proved a very real boon to nurses staying in Edinburgh or passing through. It is now about to move to much larger quarters in Drumsheugh Gardens, capable of accommodating forty nurses. The Marchioness of Linlithgow, President of the Victoria League, Edinburgh Branch, is appealing for loans of furniture for the period of the war, in order that the heavy cost of furnishing the new premises at the present time may be reduced. Every article lent will be fetched, returned, and insured by the Victoria League. It is really a good offer in these days, when extravagant rates are being paid for storing furniture and the repositories are brimming over with household effects.

The deterioration of German women under the influence of Kultur has been brought very forcibly under notice through the return of prisoners with bitter experience of their manners. It is beyond measure deplorable, but we have it continually forced under our notice, that German nurses have not escaped the foul contagion. The Bruges resident whose four years' experience of the German occupation is given in the *Times* of October 25 relates that when the hotel in which he was lodging was requisitioned for German hospital nurses the proprietors succeeded in persuading the billeting officer to find other quarters for these "horrors of war"—such was the name bestowed on nurses in Bruges! Those who had studied the type of training given to nurses in German hospitals long before the war were not altogether surprised at the quality of the finished article as evidenced in their treatment of enemy prisoners and wounded.

There are many revolting instances of the policy carried to its extreme which German doctors are known to practise in their own civilian hospitals, where the citizen who may render, if cured, good service to the State is carefully tended, while the dying are ruthlessly turned out and the aged and incurable are barely tolerated. The expulsion of the incurable women from their hospital managed by Sisters of Charity at Bruges was barbarously carried out, in spite of medical testimony that many of these poor invalids would die under removal. The sisters in charge of the lunatic asylum were compelled to make a long night journey with many violent patients in the depth of winter, during which half their bedding was stolen. The doctrine openly promulgated as regards food is typical of the depraved German morality. The extremely old and the incurable could be ignored; "they would soon die in any case." Even healthy old folk might be left to take their chance, "they had lived their lives." From this to the savage races who knock their aged parents on the head to save their keep is but a very slight step.

A movement is on foot in Canada for securing the University education of the nursing profession. The Canadian universities are already closely connected with the education of nurses, and in many directions arrangements are being made to make this connection more definite. Little can be done until peace is declared and the Army nurses are demobilised, but the university authorities are already showing themselves inclined to respond cordially to the nurses' desire for graduation. Dr. Helen M. Munday, in a paper published in the *Canadian Nurse* for September, has some remarks worth pondering over on the advantages to the nursing profession and to the community of the association of professional nursing education with university ideals, scientific equipment, and intellectual resources.

